# *TP53 Arg72Pro* and *XPD Lys751Gln* Gene Polymorphisms and Risk of Lung Cancer in Bangladeshi Patients

**DOI:** 10.31557/APJCP.2020.21.7.2091

**Published:** 2020-07

**Authors:** Tahsin Nairuz, Mostafijur Rahman, Most Umme Bushra, Yearul Kabir

**Affiliations:** 1 *Department of Biochemistry and Molecular Biology, Noakhali Science and Technology University, Bangladesh. *; 2 *Department of Biochemistry and Molecular Biology, University of Dhaka. Bangladesh. *; 3 *Department of Pharmacy, Manarat International University, Bangladesh.*

**Keywords:** Lung cancer, XPD, TP53, PCR-RFLP, genetic polymorphism

## Abstract

**Background:**

Tumor suppressor gene (*TP53*) is considered as the most frequently mutated gene in almost all forms of human cancer. Moreover, genetic variations in the *XPD* gene affect the DNA repair capacity increasing cancer susceptibility. Polymorphisms within these genes can play a major role in determining individual lung cancer susceptibility. However, several studies have investigated this possibility; but reported conflicting results. Therefore, the objective of this study was to investigate the role of* TP53 Arg72Pro* and *XPD Lys751Gln* gene polymorphisms on lung cancer susceptibility in the Bangladeshi population.

**Materials and Methods:**

Study subjects comprised of 180 lung cancer patients and 200 healthy volunteers. Genetic polymorphism of *TP53* was determined by multiplex PCR-based method, while XPD genotypes were analyzed using Polymerase Chain Reaction-based Restriction Fragment Length Polymorphism (PCR-RFLP) method. Lung cancer risk was estimated as odds ratio (OR) and 95% confidence interval (CI).

**Results:**

From the results, no significant association between *TP53 Arg72Pro* polymorphism and lung cancer risk was observed. Whereas, patients with homozygous mutant variants (Gln/Gln) of XPD at codon 751 were found significantly associated with lung cancer risk when compared to the control (OR=3.58; 95% CI=1.58-8.09; p=0.002). Lung cancer risk was found significantly higher with Gln/Gln variants of XPD among smokers (OR=4.03; 95% CI=1.11-14.63; p=0.026). Significant increased risk of lung cancer was found with Arg/Pro genotypes of TP53, Lys/Gln and Gln/Gln variants of XPD in individuals with family history of cancer (OR=3.44; 95% CI=1.36-8.72; p=0.011; OR=3.17; 95% CI=1.20-8.39; p=0.024; OR=16.35; 95% CI=0.92-289.5; p=0.007, respectively).

**Conclusion:**

The findings indicated that homozygous mutant variants (Gln/Gln) of XPD were associated with increased lung cancer risk, whereas TP53 Arg72Pro polymorphism was not associated with risk of lung cancer among Bangladeshi patients.

## Introduction

Lung cancer is one of the most prevalent malignancies with the largest number of patients all over the world. Worldwide, 2.1 million cases are newly diagnosed with lung cancer (11.6% of the total cases) and it is regarded as the leading cause of cancer death (18.4% of the total cancer deaths) predicted in 2018 (GLOBOCAN) (Bray et al., 2018). In Bangladesh, lung cancer ranks as the top prevalent cancer in males considering incidence and mortality (24.9% of incidence and 28.6% of mortality), whereas in case of females, the incidence of lung cancer (5.6%) is next to breast cancer (21.9%), cervical cancer (21.8%) and lip & oral cavity cancer (6.6%) and mortality of lung cancer (7.6%) is next to cervical (19%) and breast cancer (15.4%) (Ferlay et al., 2010). In Bangladesh, the total number of lung cancer patients was estimated to be 196,000 and 85% of those are aged 30 years or above (Hussain and Sullivan, 2013). The recent WHO data of 2017 anticipated that in Bangladesh lung cancer deaths reached 12,075 or 1.53% of total deaths (WHO, 2017). Moreover, the new cases of lung cancer in Bangladesh were recorded 14951 in 2008 and the annual number of cases is supposed to reach 43,048 by 2030 stated by IARC 2010 report (Hussain and Sullivan, 2013).

The development and progression of lung cancer are complex, multilateral, and multifactorial process. The well-established major risk factors for lung cancer are exposure to cigarette smoke and other environmental carcinogens where about 80–90% of lung cancers are attributable to cigarette smoking (Mustafa et al., 2016). Even though the carcinogenic consequences of smoking and alcohol consumption are evident, not all exposed individuals develop lung cancer which demonstrates that other factors including genetic polymorphisms may also alter the risk of this fatal disease independently or in combination with each other along with the environmental exposures (Kiyohara et al., 2006; Steliga and Dresler, 2011; Torok et al., 2011). 

The tumor suppressor gene *p53 (TP53)*, is located on chromosome 17p13, is the most frequently mutated genes in human cancer. *TP53* gene inactivation is thought to be a key contributor in the early event of lung carcinogenesis (Takahashi et al., 1989). Mutations in the *TP53* gene contribute to >90% of small cell lung cancers and >50% of non-small cell lung cancers (Nigro et al., 1989; Vousden, 2000; Yokota and Kohno, 2004). The codon 72 polymorphism (*rs1042522*) on the 4^th^ exon of the *TP53* gene was found to have an association with lung cancer (Weston et al., 1993). This polymorphism generates variant proteins with an arginine (CGC) or proline (CCC) that modifies p53 protein ability to induce apoptosis, influences the action of mutant p53, reduces DNA repair efficacy and thus may be increased the risk of lung cancer. From a meta-analysis of thirteen epidemiologic investigations, the odds ratio of lung cancer related to the Pro/Pro genotype and Pro-carrier was found 1.18 (95% CI 0.99-1.41) and was 1.02 (95% CI 0.86-1.20) respectively (Matakidou et al., 2003). In lung cancer patients, the Pro/Pro genotype has also been related to poorer prognosis and less conducive clinical outcomes (Han et al., 2008; Nelson et al., 2005; Wang et al., 1999). Furthermore, a significantly higher or lower incidence of* p53* gene mutations has been found in the Polish (Szymanowska et al., 2006) or a Norwegian population (Lind et al., 2007), respectively, among lung cancer carriers of the Pro allele. These findings highlight that corresponding to SNP and mutational status, ethnicity, and some other factors can have a great influence on p53 functionality.

Moreover, interindividual differences in lung cancer susceptibility may also be modified in part through polymorphisms in the DNA repair genes, especially those involved in the nucleotide excision repair (NER) pathway. The Xeroderma Pigmentosum Complementary group D (*XPD*) is one of the genes involved in nucleotide excision repair with an impact in p53-dependent apoptosis. *XPD* gene polymorphism at position 751 in exon 23 (*rs13181*) resulting in a lysine-to-glutamine transition, may alter the interactions of different proteins, reduce the activity of TFIIH complexes and modulate the genetic susceptibility for cancer. Several studies failed to investigate an increased risk of lung cancer with *XPD Lys751Gln *polymorphism (David-Beabes et al., 2001; Park et al., 2002), whereas other reports observed an association of lung cancer risk with variant alleles in XPD (Liang et al., 2003; Zhou et al., 2002). These inconsistent associations in previous studies might be caused by differences in study populations, possible environmental interactions, and inadequate sample sizes of earlier studies.

In Bangladesh, lung cancer ranks as the top prevalent cancer in males (Uddin et al., 2013). However, the genetic polymorphisms that are responsible for increased susceptibility to lung cancer need to be given priority to reduce the ensuing lung cancer morbidity and mortality in Bangladesh. Considering the potential association of *TP53 *and* XPD* gene polymorphisms with lung cancer, several studies have conducted in different populations. Although a limited study related to *TP53 Arg72Pro* polymorphism has been done in the Bangladeshi population, this study adds novel evidence on the prospective association of *TP53 Arg72Pro* polymorphism with a family history of lung cancer. Moreover, this is the first study on *XPD Lys751Gln* polymorphism with Bangladeshi lung cancer patients. Therefore, the objective of this study was to investigate the possible association of *TP53* and *XPD* gene polymorphisms with the risk of developing lung cancer and its aggressiveness as well as smoking impact in the Bangladeshi population.

## Materials and Methods

*Study design*

The study was designed as a case-control study with 180 lung cancer patients (cases) and 200 healthy volunteers (controls). Patients diagnosed histologically with lung cancer as per the International Association of Lung Cancer (Travis, 2011) and aged between 40 and 85 years were considered as cases and they were selected from three major cancer treatment-based hospitals in Bangladesh (Ahsania Mission Cancer and General Hospital, Dhaka Medical College Hospital, and Bangabandhu Sheikh Mujib Medical University). Controls were chosen following a physical examination and those with a previous history or any record of other severe diseases such as kidney disease, cardiovascular disease, and metastasized cancer were eliminated from the study. They were recruited from the Dhaka University Medical Centre, Department of Biochemistry and Molecular Biology, University of Dhaka, Kobi Sufia Kamal Hall, Dhaka University, Bangladesh Institute of Research and Rehabilitation in Diabetes, Endocrine and Metabolic Disorder (BIRDEM), Popular Diagnostics Center, Dhanmondi, Dhaka. All participants were explained about the purpose of the study and their written consent was taken. They filled up a structured questionnaire regarding the information on age, gender, smoking history, pathological tumor stage, and family history of chronic diseases. The protocol was approved by the Ethical Review Committees of the Department of Biochemistry and Molecular Biology, University of Dhaka, and the study was carried out according to the declaration of Helsinki and its subsequent revisions (WMA, 2013).

*Sample collection*

After taking necessary aseptic precautions approximately 3.0 mL venous blood was collected from all cases and controls with a disposable syringe. The collected sample was immediately transferred to EDTA containing tube (1.20 mg/mL) and then transported to the laboratory by keeping in an icebox. Until DNA extraction, the blood samples were refrigerated at −20°C.

*DNA extraction and Genotyping*

**Figure 1:**
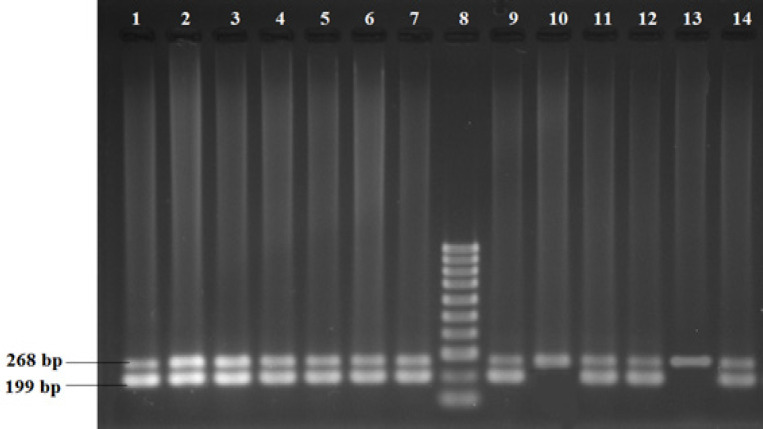
Multiplex-PCR Analysis of Arginine Specific Exon of *TP53* Gene Polymorphism. Arginine specific exon of *TP53 (*199bp), internal control β-globin (268bp). Lane 1-7,9,11,12,14 indicates the presence of arginine specific exon of the TP53 gene. Lane 10,13 represents individuals who are negative for this specific exon of the *TP53 *gene. Lane 8 indicates the molecular marker of the 100 bp ladder

**Figure 2:**
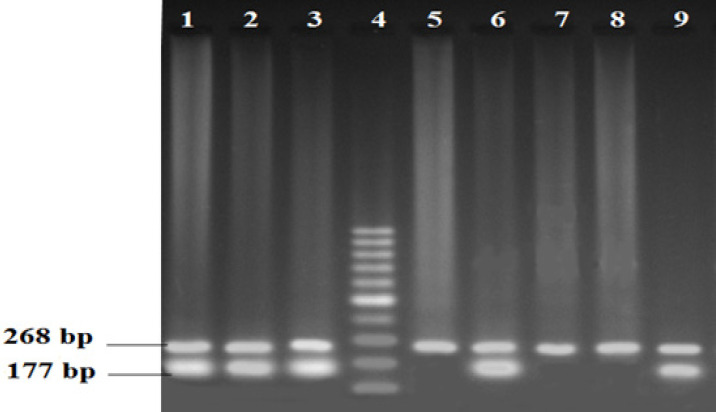
Multiplex-PCR Analysis of Proline Specific Exon of *TP53* Gene Polymorphism. Proline specific exon of *TP53* (177bp), internal control β-globin (268bp). Lane 1-3,6,9 indicates the presence of proline specific exon of the *TP53* gene. Lane 5,7,8 represents individuals who are negative for this specific exon of the *TP53* gene. Lane 4 indicates the molecular marker of the 100 bp ladder

**Figure 3:**
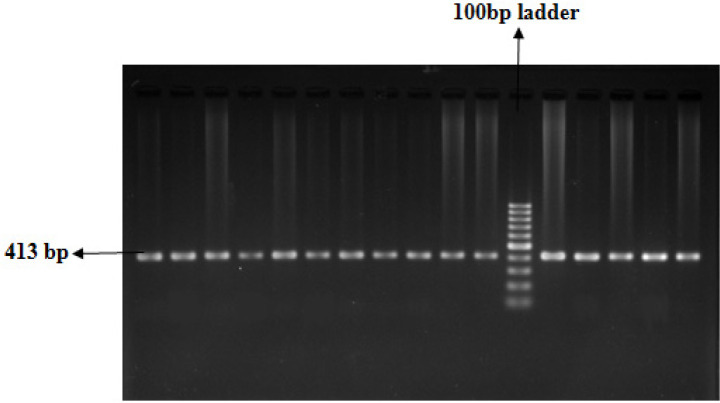
Representative PCR Products of the *XPD* Gene in 2% Agarose Gel

**Figure 4.**
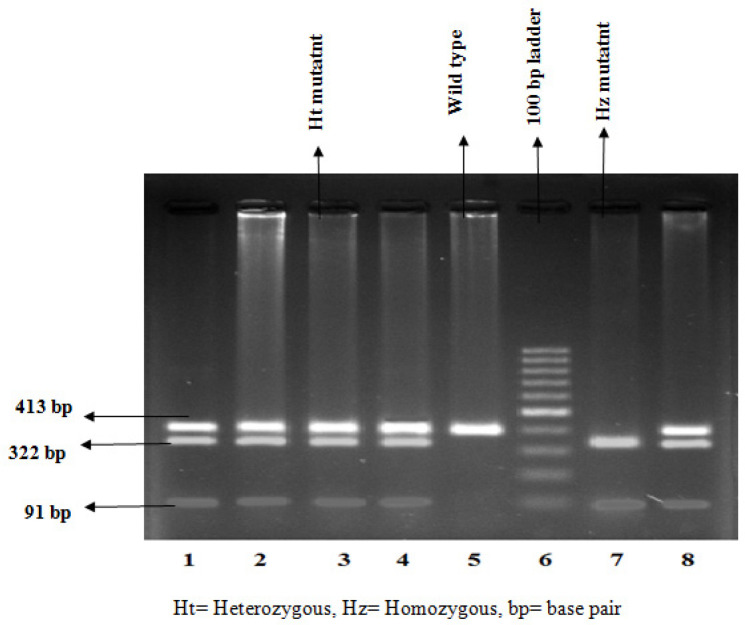
Representative Picture of Digestion of *XPD* by PstI in 3% Agarose Gel

The extraction of genomic DNA from blood samples was done through the organic extraction procedure described by Bailes et al., (2007). Genotyping of TP53 was analyzed by allele-specific multiplex PCR assay which selectively identifies the presence of either Arg or Pro p53 allele. The PCR assay was performed individually for each of the two polymorphic alleles.* β-globin* gene was amplified in both reactions as an internal positive control for ensuring successful PCR. On the other hand,* XPD* genotyping was done by PCR-Restriction Fragment Length Polymorphism (RFLP) method. The PCR conditions and primer sequences were used from the previously published papers (Mitra et al., 2009; Paoadakis et al., 2002). The co-amplified PCR products of *TP53* were resolved in 2% agarose gel following ethidium bromide staining, whereas that of *XPD* was analyzed with RFLP using the restriction enzyme PstI. The optional size of the product was ascertained by comparing it with the DNA ladder. For TP53, the Arg allele and Pro allele were determined by the presence of 199 bp and 177 bp PCR amplified products respectively (Figures 1 and 2). For the XPD genotype, fifteen microliters of PCR product (413 bp) were digested at 37^o^C for 16 hours in a water bath with the respective restriction enzyme. The enzyme digestion product was analyzed on 3% agarose gel and visualized using a gel documentation system following ethidium bromide staining (Figure 3). Upon restriction digestion, the homozygous wild type (AA) genotype produced a single band of 413 bp, the homozygous mutant genotype (CC) generated two bands of 322 bp and 91 bp, and the heterozygous genotype (AC) indicated three bands of 413 bp, 322 bp and 91 bp (Figure 4). 

*Statistical analysis*

Statistical analyses were done through SPSS Windows version 24. The relative association between cases and controls was measured by calculating the odds ratio (OR). By using logistic regression models OR, as a measure of relative risk, were estimated at 95% confidence intervals (95% CI). Fisher’s and chi-square tests were assessed by GraphPad Prism, version-7. A p-value of less than 0.05 was regarded as a level of significance.

## Results

*Characteristics of the study subjects*

The baseline characteristics of the study subjects according to age, gender, smoking status, family history of cancer are presented in Table 1. Briefly, no significant differences were found in the mean age between the two groups. On the other hand, the result of demographic data shows significant differences in gender, smoking status, and family history of cancer among study subjects (p<0.001). Present and ex-smokers were regarded as ever smokers, whereas persons who had never smoked at the periods of his/her lifetime were considered as never smokers.

*Frequency distribution of TP53 and XPD genotype and risk of lung cancer*

The frequency distribution of TP53 Arg72Pro and XPD Lys751Gln genotypes in control and patient with their estimated risk of lung cancer is presented in Table 2. In lung cancer patients, the frequency of arginine homozygous (Arg/Arg), proline homozygous (Pro/Pro), and arginine/proline heterozygous (Arg/Pro) was found 37%, 5% & 58% respectively. Whereas, in controls, the frequency of arginine homozygous (Arg/Arg), proline homozygous (Pro/Pro) and arginine/proline heterozygous (Arg/Pro) was 36.67%, 7.22 % & 56.11% respectively. No significant differences were present in the frequency distribution of proline homozygous (Pro/Pro) (OR= 0.976, 95% CI= 0.64-1.50, p=0.501) and arginine/proline heterozygous (Arg/Pro) (OR= 1.458, 95% CI= 0.60-3.55, p=0.914) between controls and lung cancer patients, when Arg/Arg was considered as reference group. 

On the other hand, among 180 patients of lung carcinoma, homozygous wild type (Lys/Lys) was 44.44%, heterozygous mutant variant (Lys/Gln) was 41.67% and homozygous mutant variant (Gln/Gln) was 13.89%. Whereas in controls, 51.50% was homozygous wild type (Lys/Lys), 44% was heterozygous mutant variant (Lys/Gln) and 4.50% was homozygous mutant variant (Gln/Gln). Patients with homozygous mutant variants (Gln/Gln) of XPD at codon 751 were found significantly associated with lung cancer risk when compared to the control (OR=3.58; 95% CI=1.58-8.09; p=0.002), whereas no significant association was found with heterozygous mutant variants (Lys/Gln) (OR=1.10; 95% CI=0.72-1.68; p=0.745) when Lys/Lys was considered as a reference group. 

*TP53 and XPD genotype on risk of lung cancer according to smoking status and family history of lung cancer*

Association of TP53 and XPD genotypes and lung cancer risk according to smoking status and family history are shown in Table 3. The risk of developing lung cancer was low for both smokers and nonsmokers with Arg/Pro genotype. Whereas, the frequency of Pro/Pro genotype was found nonsignificantly higher (OR=2.88; 95% CI=0.64-12.87; p>0.05) in nonsmoker lung cancer patients compared to control. In the case of XPD, lung cancer risk was low for both smokers and nonsmokers with Lys/Gln genotype. Whereas, the frequency of Gln/Gln genotype was significantly higher (OR=4.03; 95% CI=1.11-14.63; p=0.026) in smoker lung cancer patients compared to control.

In the case of a family history of cancer, the frequency of the Arg/Pro genotype was found significantly higher in lung cancer patients having a family history of cancer compared to control (OR=3.44, 95% CI=1.36-8.72, p=0.011). On the other hand, none of the variants were found significantly associated with lung cancer risk in case of having no family history of cancer. On the other hand, the frequency of Lys/Gln and Gln/Gln variants of XPD was significantly higher in cancer patients having a family history of cancer compared to control (OR=3.17; 95% CI=1.20-8.39; p=0.024; OR=16.35; 95% CI=0.92-289.5; p=0.007 respectively). None of the variants were found significantly associated with lung cancer risk in case of having no family history of cancer.

In this study, the frequencies of TP53 and XPD genotype in the lung cancer patients according to tumor stage IIIA and IIIB (Lung cancer stage group accordance with TNM classification) (Liu and Zhi, 2015) were also analyzed but no significant association of these polymorphisms with tumor stages was observed. Similarly, the analysis of pairwise joint associations of TP53 Arg72Pro and XPD Lys751Gln genotypes with lung cancer risk showed the statistically non-significant result, although the odds ratios of combined mutant or deficient variants of the studied genes were higher in lung cancer patients than controls.

**Table 1:** Baseline Characteristics of the Study Subject

Variable	Control (n=200) n (%)	Patient (n=180) n (%)	*P*-value
Age (Years)			
<50	66 (33.00)	43 (23.88)	
50-70	129 (64.50)	126 (70.00)	
>70	5 (2.50)	11 (6.11)	
Age (Mean±SEM)	54.70±0.55	55.83±0.66	ns
Gender			
Male	155 (77.50)	109 (60.56)	
Female	45 (22.50)	71 (39.44)	<0.001
Smoking status			
Never	125 (62.50)	52 (28.89)	
Ever	75 (37.50)	128 (71.11)	<0.001
Family history of cancer			
Yes	28 (14.00)	85 (47.22)	
No	172 (86.00)	95 (52.78)	<0.001

Fisher’s and chi-square tests were done to calculate the statistical significance; p<0.05 considered as a level of significance; ns, not significant

**Table 2:** Genotypic Distribution of *TP53 *(codon 72) and *XPD* (codon 751) in the Study Subject and Risk of Lung Cancer

Gene	Genotype	Control (n=200) n (%)	Patient (n= 180) n (%)	OR (95% CI)	*P*-value
*TP53*	Arg/Arg	74 (37.00)	66 (36.67)	1.0 (Ref.)	
	Arg/Pro	116 (58.00)	101 (56.11)	0.976 (0.64-1.50)	0.914
	Pro/ Pro	10 (5.00)	13 (7.22)	1.458 (0.60-3.55)	0.501
*XPD*	Lys/Lys	103 (51.50)	80 (44.44)	1.0 (Ref.)	
	Lys/Gln	88 (44.00)	75 (41.67)	1.097 (0.72-1.68)	0.745
	Gln/Gln	9 (4.50)	25 (13.89)	3.576 (1.58-8.09)	0.002*

Odds ratios (OR) and 95% confidence interval (95%CI); *p<0.05 considered as level of significance.

**Table 3:** *TP53* and *XPD* Genotype on Risk of Lung Cancer According to Smoking Status and Family History of Cancer

Smoking Status/ Family History of Cancer	Gene	Genotype	Control (n=200)	Patient (n=180)	OR (95% CI)	*P*- value
		Arg/Arg	28	50	1.0 (Ref.)	-
Smoker		Arg/Pro	41	69	0.94 (0.52-1.72)	ns
		Pro/ Pro	6	9	0.84 (0.27-2.61)	ns
	*TP53*					
		Arg/Arg	46	16	1.0 (Ref.)	-
Non Smoker		Arg/Pro	75	32	1.23 (0.61-2.48)	ns
		Pro/ Pro	4	4	2.88 (0.64-12.87)	ns
						
		Lys/Lys	39	58	1.0 (Ref)	-
Smoker		Lys/Gln	33	52	1.06 (0.58-1.92)	ns
		Gln/Gln	3	18	4.03 (1.11-14.63)	0.026*
						
	*XPD*	Lys/Lys	64	22	1.0 (Ref)	-
		Lys/Gln	55	23	1.22 (0.61-2.42)	ns
Non Smoker		Gln/Gln	6	7	3.39 (1.03-11.19)	ns
						
						
		Arg/Arg	17	28	1.0 (Ref.)	-
Yes		Arg/Pro	9	51	3.44 (1.36-8.72)	0.011*
		Pro/ Pro	2	6	1.82 (0.33-10.07)	ns
	*TP53*					
No		Arg/Arg	57	38	1.0 (Ref.)	-
		Arg/Pro	107	50	0.70 (0.41-1.19)	ns
		Pro/ Pro	8	7	1.31 (0.44-3.92)	ns
		Lys/Lys	21	35	1.0 (Ref.)	-
Yes		Lys/Gln	7	37	3.17 (1.20-8.39)	0.024*
		Gln/Gln	0	13	16.35 (0.92-289.5)	0.007*
	*XPD*					
No		Lys/Lys	82	45	1.0 (Ref.)	-
		Lys/Gln	81	38	0.85 (0.50-1.45)	ns
		Gln/Gln	9	12	2.43 (0.95-6.21)	ns

Odds ratios (OR) and 95% confidence interval (95%CI); *p<0.05 considered as level of significance; ns, not significant

## Discussion

This population-based case-control study was carried out to evaluate the association of Tumor suppressor gene *p53 (TP53)* and Xeroderma Pigmentosum Complementary Group D *(XPD)* gene polymorphisms on lung cancer susceptibility in the Bangladeshi population. Worldwide, numerous studies investigating the role of *TP53* and* XPD* gene polymorphisms with lung cancer have prompted to inconsistent reports, even though several studies have identified that individuals polymorphic for the tumor suppressor or DNA repair genes are increased the risk for developing lung cancer. For this study, we had undertaken 180 lung cancer patients and 200 healthy controls where both patients and healthy controls belonged to the same ethnic background and all shared a common geographic origin. The results of basic demographic data showed significant differences in gender, smoking status, and family history of cancer (Table 1).

The distribution of the three genotypes of TP53, Arg/Arg, Arg/Pro, and Pro/Pro was found 36.67%, 56.11% and 7.22% in lung cancer patients compared to 37%, 58% and 5% in healthy control respectively (Table 2). These results indicated no statistically significant association of Arg/Pro or Pro/Pro genotype with increased risk of developing lung cancer when Arg/Arg was considered as a reference group, that is consistent with the results reported by Jung et al. (2008) and Mechanic et al. (2007). Although, Chowdhury et al. (2015) and Mostaid et al. (2014) noted a significant association of *TP53 Arg72Pro* gene polymorphism with lung cancer susceptibility in the Bangladeshi population, the inconsistency result between our study with that of previous two studies may be related to the sample size, differences in histological type and clinical stages of lung cancer as well as the age of the study subjects (cancer patients) between their study populations (Chowdhury et al., 2015; Mostaid et al., 2014) and ours. 

In contrast to tumor suppressor gene *TP53*, a significant association was found between DNA repair gene *XPD Lys751Gln* polymorphism and risk of lung cancer. Patients with homozygous mutant variants (Gln/Gln) of XPD were found significantly associated with a 3.6 fold increased risk of lung cancer compared to the control (OR=3.58; 95% CI=1.58-8.09; p=0.002), whereas no significant association was found with heterozygous mutant variants (Lys/Gln) (Table 2). These findings are compatible with a previous case-control study that found a significant association of *XPD Lys751Gln* gene polymorphism with an increased risk of developing lung cancer (Zhou et al., 2002; Liang et al., 2003). Whereas, several studies failed to identify an association of the *XPD Lys751Gln* polymorphism with lung cancer (David-Beabes et al., 2001; Park et al., 2002). The inconsistent results in several studies for the *XPD *polymorphisms might be attributable to differences in ethnicities, selected study subjects, the small sample size, and probable environmental interactions.

Exploring the association of smoking status, tumor stage, and family history of cancer with genotypes and lung cancer risk, no significant increased risk of lung cancer was found in smokers in the case of TP53 genotypes (Table 3). This result is consistent with that reported by Wang et al. (2013). Kawajiri et al. also anticipated that *p53* polymorphism modifies smoking-induced lung cancer risk independently of other genetic risk factors (Kawajiri et al., 1993). In the case of *XPD Lys751Gln* gene polymorphism, the frequency of the Gln/Gln variant significantly higher was found in smoker lung cancer patients compared to nonsmokers (Table 3). Similarly, Zhou et al. found that smoking could modulate the impacts of *XPD* gene polymorphisms on lung cancer risk, suggesting a gene–environmental influence in lung carcinogenesis (Zhou et al., 2003).

Significant increase risk of lung cancer with Arg/Pro genotype of TP53, Lys/Gln and Gln/Gln variants of XPD was found in individuals with a family history of cancer (Table 3), which revealed that family history of cancer may be a risk factor for patients having these gene variants. On the contrary, no significant association of *TP53 *(codon 72) and *XPD* gene polymorphisms was observed with stage IIIA and IIIB lung cancer patients which signified that there was no association between these genotypes and the frequency of more aggressive tumor. 

Considering that different combined polymorphisms were related to a more increased risk of cancer, the *TP53* and *XPD* genotype combinations were also analyzed in this study for correlations with the increased risk of lung cancer, but these results are not statistically significant. The lack of statistical significance in this study may be due to the selection of different pathway genes that are not interrelated or inadequate sample size. 

In conclusion, our study suggested a prospective association of genetic polymorphisms with increased susceptibility to lung cancer. The study revealed that *Gln/Gln* variants at codon 751 of *XPD* are associated with the susceptibility of lung cancer, whereas *TP53 Arg72Pro* gene polymorphism is not associated with increased risk of developing lung cancer in Bangladeshi population. Considering the genetic backgrounds of the study subjects, the results may be attributed to taking an individualized therapeutic decision in lung cancer. Nevertheless, this study had a small sample size hence, further studies with a large sample size are needed to determine the true impact of genetic susceptibility in lung cancer in Bangladesh. 

Authors’ Contribution: Tahsin Nairuz designed and implemented the study, performed data analysis, and drafted the paper. Md. Mostafijur Rahman assisted in implementation and data analysis. Most Umme Bushra follows up patients’ enrollment, helps in sample collection and data management. Yearul Kabir provided overall guidance and support to the study and critically reviewed the manuscript. All authors have read and approved the final manuscript. None of the authors had a personal or financial conflict of interest.
